# The risk of selenium deficiency in Malawi is large and varies over multiple spatial scales

**DOI:** 10.1038/s41598-019-43013-z

**Published:** 2019-04-25

**Authors:** Felix P. Phiri, E. Louise Ander, Elizabeth H. Bailey, Benson Chilima, Allan D. C. Chilimba, Jellita Gondwe, Edward J. M. Joy, Alexander A. Kalimbira, Diriba B. Kumssa, R. Murray Lark, John C. Phuka, Andrew Salter, Parminder S. Suchdev, Michael J. Watts, Scott D. Young, Martin R. Broadley

**Affiliations:** 1 School of Biosciences, University of Nottingham, Sutton Bonington Campus, Loughborough, Leicestershire, LE12 5RD UK; 2 Department of Nutrition, HIV and AIDS, Ministry of Health, Lilongwe, Malawi; 3 Inorganic Geochemistry, Centre for Environmental Geochemistry, British Geological Survey, Nottingham, NG12 5GG UK; 4 Community Health Sciences Unit, Ministry of Health, Private Bag 65, Lilongwe, Malawi; 5 The Department of Agricultural Research Services, P.O. Box 30799, Lilongwe 3, Malawi; 6 Faculty of Epidemiology and Population Health, London School of Hygiene & Tropical Medicine, Keppel Street, London, WC1E 7HT UK; 7 Department of Human Nutrition and Health, Faculty of Food and Human Sciences, Bunda Campus, Lilongwe University of Agriculture and Natural Resources, P.O. Box 219, Lilongwe, Malawi; 8 School of Public Health and Family Medicine, College of Medicine, University of Malawi, Private Bag 360, Chichiri, Blantyre 3 Malawi; 9 Department of Pediatrics and Hubert Department of Global Health, Emory University, Atlanta, Georgia 30322 USA

**Keywords:** Geochemistry, Predictive markers

## Abstract

Selenium (Se) is an essential human micronutrient. Deficiency of Se decreases the activity of selenoproteins and can compromise immune and thyroid function and cognitive development, and increase risks from non-communicable diseases. The prevalence of Se deficiency is unknown in many countries, especially in sub-Saharan Africa (SSA). Here we report that the risk of Se deficiency in Malawi is large among a nationally representative population of 2,761 people. For example, 62.5% and 29.6% of women of reproductive age (WRA, n = 802) had plasma Se concentrations below the thresholds for the optimal activity of the selenoproteins glutathione peroxidase 3 (GPx3; <86.9 ng mL^−1^) and iodothyronine deiodinase (IDI; <64.8 ng mL^−1^), respectively. This is the first nationally representative evidence of widespread Se deficiency in SSA. Geostatistical modelling shows that Se deficiency risks are influenced by soil type, and also by proximity to Lake Malawi where more fish is likely to be consumed. Selenium deficiency should be quantified more widely in existing national micronutrient surveillance programmes in SSA given the marginal additional cost this would incur.

## Introduction

More than 800 million people have insufficient dietary energy intake globally, with >2 billion people at risk of one or more micronutrient deficiencies (MNDs), or *Hidden Hunger*^1^. Global disease burdens attributed to MNDs are based on iron (Fe), zinc (Zn), iodine (I) and vitamin A, which are considered to be the MNDs of greatest public health concern by the World Health Organization (WHO)^1^. However, recent estimates of dietary mineral supplies, derived from Food Balance Sheets (FBS) combined with food composition tables, indicate that other MNDs, such as calcium (Ca) and selenium (Se) may also have considerable public health significance at a regional level^2–6^.

Selenium has many roles in human health. Deficiency of Se, which is typically due to inadequate dietary Se intake, decreases the expression and activity of several essential selenoproteins and this has been associated with reduced immune function, thyroid function and cognitive development, and increased risks from non-communicable diseases^7–9^. A high prevalence of inadequate dietary Se has been inferred from FBS for many countries in sub-Saharan Africa (SSA)^3^. In Malawi, an estimated 70% of the population consumed inadequate Se based on nationally-representative household-level food consumption data^6^. Small-scale cross-sectional studies of food intakes^10,11^, composite diets^10^, and Se status in blood plasma among the Malawi population are consistent with predictions that the majority of the Malawian population are Se deficient^10,12^.

We tested if Se deficiency is prevalent among the Malawian population, using blood plasma Se as an accepted population biomarker of Se status^7–9^. Plasma samples were collected during the previously planned Malawi Micronutrient Survey (MNS) and Malawi Demographic and Health Survey (MDHS) of 2015-16^13^. The MNS comprised a nationally representative sample of preschool children (PSC, aged 6–59 months), school-aged children (SAC, aged 5–14 years), women of reproductive age (WRA, aged 15–49 years), and men (aged 20–54 years). The scope of the MNS/MDHS, including details of the sampling design, ethics, informed consent, and individual confidentiality procedures are described in the Methods. The Se concentration of plasma was determined using inductively coupled plasma-mass spectrometry (ICP-MS). In total, data were obtained for 2,761 individuals from 1,233 households, located in 102 clusters, 84 and 18 of which were located in rural and urban areas, respectively. Data were analysed using a linear mixed model to quantify long- and short-range spatial variation in Se status^14^. Conditional distributions of Se status could be computed from this model at unsampled sites and, from these, both the expected status and the probabilities that plasma Se concentration of WRA was <84.9 ng mL^−1^ and <64.8 ng mL^−1^. These threshold values were defined based on optimal activities of the selenoproteins glutathione peroxidase 3 (GPx3) and iodothyronine deiodinase (IDI), respectively, in adults^9^. These probabilities were presented as maps, using a verbal scale to visualise the probability that plasma Se falls below the thresholds of interest.

## Results and Discussion

### Prevalence of selenium deficiency

Selenium deficiency is widespread among the Malawi population. Across all demographic groups, the mean and median plasma Se concentration was 73.2 ng mL^−1^ and 68.2 ng mL^−1^, respectively (standard deviation, SD, 33.9 ng mL^−1^; range 9.9–374 ng mL^−1^; Table 1). Plasma Se concentration increased with age, ranging from a median of 57.7 ng mL^−1^ (±26.2 ng mL^−1^ SD) in PSC, to 81.9 ng mL^−1^ (±27.9 ng mL^−1^ SD) in adult men (Table 1). To estimate Se deficiency prevalence, we determined the proportion of WRA and men whose plasma Se concentration was less than thresholds previously established for the optimal activities of two essential selenoproteins, GPx3 and IDI, involved in oxidative stress response and thyroid function, respectively^9^. Plasma Se concentration was below the threshold for optimal GPx3 activity in 62.5% of WRA and 57.2% of men (Table 2). Plasma Se concentration was below the threshold for optimal IDI activity in 30% of WRA and 22% of men. Whilst thresholds for the optimal activity of GPx3 and IDI were used here to indicate Se deficiency, other selenoproteins, including Selenoprotein P, whose optimal activity is at blood plasma Se concentration >100 ng mL^−1 ^^7^, could be used for this type of analysis and would indicate a much greater overall deficiency prevalence. Thus, further research is required to identify the most suitable threshold for a particular geographical and demographic context^7^. The subsequent spatial statistical analysis in this study focused on WRA, due to the larger sample size and accepted deficiency thresholds for adults. However, if we assume the same thresholds of deficiency among children as for adults, then 86% and 64% of PSC are at risk of suboptimal expression of GPx3 and IDI, respectively. Given the potential implications of Se deficiency for immune function, thyroid function, and cognitive development, there is an urgent need to establish thresholds for Se deficiency in children.

**Table 1:** Plasma selenium (Se) concentration among demographic groups in Malawi.

Characteristic	n	Median	Mean	SD	Min	Max
		ng mL^−1^				
**PSC**						
Females	494	57.5	60.9	25.1	9.9	207
Males	496	57.7	63.4	27.3	12.5	198
**SAC**						
Females	385	69.2	74.6	37.4	11.0	284
Males	369	67.1	72.5	31.7	17.6	281
**WRA**	802	78.4	83.7	38.4	11.1	374
**Men**	215	81.9	83.3	27.9	17.5	198
**All**	2761	68.2	73.2	33.9	9.9	374

n = sample number in cohort; SD = standard deviation; Min = minimum value in the cohort; Max = maximum value in the cohort; PSC = preschool children; SAC = school-aged children; WRA = women of reproductive age (aged 15–49 years); Men (aged 20–54 years).

**Table 2:** Prevalence of selenium (Se) deficiency among women of reproductive age (WRA, aged 15–49 years) and men (aged 20–54 years) in Malawi, based on plasma Se concentration thresholds for optimal activity of glutathione peroxidase 3 (GPx3; <84.9 ng mL^−1^), iodothyronine deiodinase (IDI; <64.8 ng mL^−1^), and for Keshan Disease (KD; <30 ng mL^−1^) which is a cardiomyopathy linked to Se status reported in China. Thresholds from Thomson (ref.^9^).

Characteristic		n	Prevalence (%) below the plasma Se concentration threshold		
			GPx3	IDI	KD
**WRA**	Urban	126	52.3	12.7	0.0
	Rural	676	64.3	32.7	1.8
	All	802	62.5	29.6	1.5
**Men**	Urban	28	50.0	17.9	0.0
	Rural	187	58.3	23.0	1.1
	All	215	57.2	22.3	0.9

### Spatial determinants of Se status

**Figure 1:**
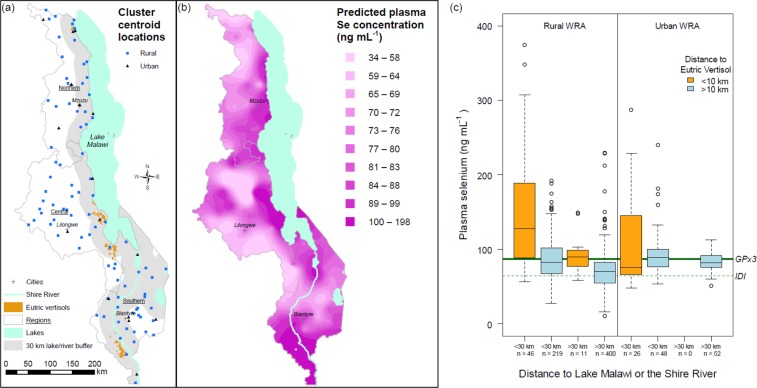
Selenium (Se) status of the Malawi population: (**a**) location of rural and urban Enumeration Areas from which study participants were recruited, (**b**) median unbiased predicted plasma Se concentration of women of reproductive age (WRA), (**c**) plasma Se concentration among WRA according to proximity to Eutric Vertisol soil classification, and to Lake Malawi or the Shire River; the threshold for the optimal activity of iodothyronine deiodinase (IDI) and glutathione peroxidase 3 (GPx3) are indicated by dashed and solid lines, respectively.

A much greater proportion of the variation in plasma Se concentration arose between clusters than within clusters, showing that Se status is under strong geospatial control (Fig. 1). The variation between cluster means shows spatial dependence up to a distance of 120 km. We can hypothesise that a spatial dependency in Se status among the Malawi population is due primarily to variation in maize grain Se concentration and subsequently dietary Se supplies. In Malawi, maize represented 48% of dietary energy supply in 2013^15^ and contributed ~70% of energy consumption in the poorest quintile of households^6^. Food production and consumption is highly localised in Malawi, e.g. in the 2011 Third Integrated Household Survey (IHS3) it was reported that 57% of maize flour consumed by households came from subsistence production, while the remainder (38% from purchases and 5% from gifts) was likely be sourced locally in rural locations^6,16^. The consumption of foods known to be good sources of Se (e.g. animal products including fish) is limited in Malawi although is greater in populations living closer to Lake Malawi, other lakes, and the Shire River^6,8,16^. Whilst strong links between the Se concentration in food crops, dietary Se intakes, and Se status have previously been reported in small cross-sectional studies in Malawi^10^, more research is needed to establish the relative contribution of different plant and animal sources to Se intake and status, including bioavailability studies, at a range of geographical scales.

Plasma Se concentrations were much greater in populations living in proximity to vertisol soil types in the south and east of Malawi, and in populations living closer to Lake Malawi and the Shire River (Fig. 1). For example, plasma Se concentrations among rural dwelling WRA living within 10 km of the nearest mapped vertisol, and within 30 km of the lake or river, were 148 ± 76 ng mL^−1^ (mean ± SD; n = 46). Vertisol soils in Malawi are typically located close to Lake Malawi and the Shire River, where fish consumption are likely to be greater. However, the main effect driving Se intakes and status is likely to be soil factors. Notably, the greatest plasma Se concentrations among WRA were observed in Salima District, an area next to the lake where vertisol soils also occur. In Salima, the plasma Se concentration of some individual WRA exceeded 300 ng mL^−1^ (range 104–374 ng mL^−1^; n = 11). These observations are consistent with a previous cross-sectional study that showed much greater plasma Se concentration of 119 ± 23 ng mL^−1^ (mean ± SD; n = 60) among WRA living close to vertisol soils in Mikalango Extension Planning Area (EPA), compared to 54 ± 10 ng mL^−1^ (mean ± SD; n = 60) for those living in Zombwe EPA^10^. Previously, the Se concentration of maize grain growing on vertisol soils in Malawi was shown to be 10–15-fold greater than in grain grown on acidic soils which dominate Malawi croplands^2^. In acidic tropical soils, Se is largely unavailable to plants probably due to its strong adsorption to iron and aluminium oxides as Se^(IV)^. In vertisol soils, which cover ~0.5% of the land area in Malawi, Se is more readily available to plants because Se^(IV)^ forms are adsorbed less strongly at higher soil pH. Furthermore, Se^(VI)^ forms are more stable at higher soil pH under aerobic soil conditions and are taken up more efficiently than Se^(IV)^ by plant roots^17^.

**Figure 2:**
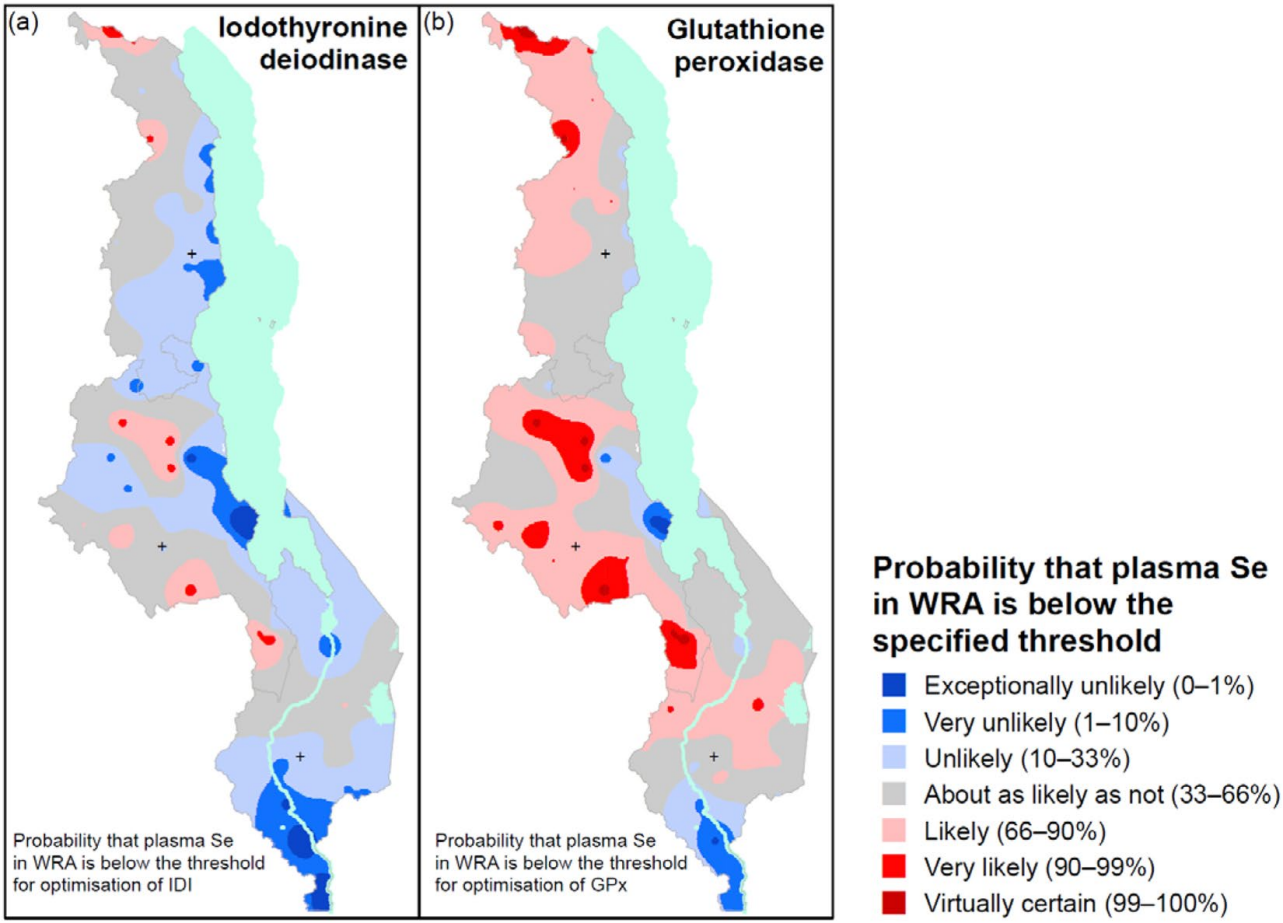
Probability that measured plasma selenium (Se) concentration of women of reproductive age (WRA) would fall below the threshold for the optimal activity of (**a**) iodothyronine deiodinase (IDI), (**b**) glutathione peroxidase 3 (GPx3). The legend units are defined verbally, and by a range of probability values, expressed as percentages.

The output from this spatial analysis allows us to visualise the probability of Se deficiency at a national scale, using a verbal scale that shows the likelihood of an individual being below a threshold (Fig. 2). These maps and the underlying statistical model could be used to plan further investigations. For example, one might plan future national-scale sampling so as to reduce uncertainties in the predictions where these are large, and one might optimise the balance of sample effort between the inclusion of new clusters or the amount of replication at household or within-household level.

### Socioeconomic determinants of Se status

The prevalence of Se deficiency was associated with household wealth. Plasma Se concentration was below the threshold for optimal GPx3 activity among 70%, 68%, 59%, 59% and 56% of WRA from households in the poorest to wealthiest quintiles, respectively. These trends are qualitatively consistent with estimates of inadequate dietary Se supplies among 92%, 86%, 81%, 72% and 51% of households from the poorest to wealthiest quintiles, respectively, based on household-level food consumption and spatially disaggregated food composition data^6^. The prevalence of Se deficiency among WRA in urban areas was lower than in rural areas, i.e. 34% and 62%, respectively, were below the threshold for optimal GPx3 activity. Households in urban areas typically had greater expenditure on food^16^, while urban markets are likely to provide more diverse foods of wider geographical origin.

### Implications for micronutrient surveillance programmes and policies

There is evidence that Se status is under spatial control in other countries in SSA, albeit based on much smaller cross-sectional geographical studies, including Cote d’Ivoire, the Democratic Republic of the Congo, and Ethiopia^18–23^. Taken together, these data indicate that prevalence of Se deficiency is likely to be widespread in SSA^3^, and that the variability at subnational scales needs to be explored and communicated at finer resolution, using ‘*GeoNutrition’* type spatial frameworks as described in this study. Such frameworks also need to include the collection of disaggregated data on the Se (and other mineral) supply characteristics of soils and food crops. These data are not yet available in the same form as the plasma Se concentration data reported in this study^2,5,6^. Conducting a MNS is expensive, time-consuming and logistically-challenging. Given that population Se deficiency can be inferred readily from plasma Se concentration data, there is a strong case for adapting MNS in SSA to quantify Se deficiency given that no additional burden is placed on participants. There is also a clear need to establish thresholds for Se deficiency in children, especially given the evidence that children are less likely than adults to have access to foods that are good sources of Se within households^24^.

The present study provides the strongest evidence to-date of widespread Se deficiency in a national population in SSA. The prevalence of Se deficiency is likely to remain high in Malawi without a public health intervention. There is scope to alleviate Se deficiency through increasing dietary diversity. Thus, whilst Se deficiency is prevalent in all socio-economic groups, the risk of Se deficiency is lower amongst wealthier groups and increased consumption of animal products is one dietary diversification option. However, unless animals have access to Se supplements in feeds, the concentration of Se in livestock products, as well as livestock productivity and health, will be constrained by low concentrations of Se in forages due to soil factors. Leaves of the tree crop *Moringa oleifera* and *M. stenopetala* (Moringaceae) have been shown to be a good potential dietary source of Se across a range of soil types from surveys in Malawi^5^, Kenya^25^ and Ethiopia^25^. Agronomic biofortification (agro-fortification) of maize using Se-enriched fertilisers has been proposed as a cost-effective strategy to alleviate Se deficiency in Malawi^2,6,26,27^; such an approach has been adopted successfully in Finland since 1984^28^. The ethics, efficacy, effectiveness, and safety of interventions to promote dietary diversification and/or micronutrient fertilisers would, of course, need to be assessed carefully by national policy makers in the context of other public health spending.

## Methods

### Aim of study

The aim of the study was to determine Se status of individuals at a nationally representative scale in Malawi, using blood plasma Se concentration as a population-level biomarker. The study was conducted using plasma samples and data collected during the Malawi Micronutrient Survey (MNS) and Demographic and Health Survey (MDHS) of 2015-16^13,29^. The wider purpose of the MNS was to provide a range of data for planning national nutritional policies. The study comprised a nationally representative sample of preschool children (PSC, aged 6–59 months), women of reproductive age (WRA, aged 15–49), school-aged children (SAC, aged 5–14), and men (aged 20–54). Details of the scope of the full MNS, including details of the design, informed consent, and individual confidentiality procedures are described elsewhere^13,29,30^. The study was conducted under informed consent. Ethical approval for blood sampling and testing for mineral micronutrient biomarkers as part of the MNS was granted by the Malawi National Health Sciences Research Committee (NHSRC), reference number NHSRC 15/5/1436. Because the MNS had not originally planned to analyse blood samples for Se, a material transfer agreement (MTA) linked to NHSRC 15/5/1436 was considered and approved by the NHSRC (11^th^ January 2016), to include Se as an additional mineral micronutrient biomarker. There were no modifications to the MNS sample collection procedures. All methods were performed in accordance with the relevant guidelines and regulations.

### Sampling

The MNS represented a subsample of the wider MDHS. The MDHS was designed as a cross-sectional study, with a two-stage cluster sampling design to enable indicators to be obtained nationally and for each of the 28 Districts of Malawi, for both urban and rural populations. The sampling frame was based on the Malawi Population and Housing Census of 2008. For the first stage of the MDHS, a total of 850 Enumeration Areas (EAs), referred to as clusters, were selected from a total of >9,000 EAs in Malawi using a probability proportional to population size. For the second stage of the MDHS, 30 and 33 households were selected at random from each urban and rural cluster, respectively, using an updated list of the eligible 27,531 households within the 850 MDHS clusters.

For the MNS, 105 target clusters were selected randomly from the MDHS clusters so that 8 urban and 27 rural clusters were selected from each region (North, Central and South); a total target of 24 and 81 urban and rural clusters, respectively. From each of the urban and rural MNS clusters, 10 and 11 households, respectively, were excluded from the MNS because these households had been selected for HIV testing within the MDHS. Therefore, the MNS comprised 20 and 22 households from each urban and rural cluster, respectively, giving a target sample size of 480 and 1782 urban and rural households, respectively.

Within each of the households selected for the MNS, eligible participants (defined as having spent the night prior to that survey in that household) were invited to participate as follows: PSC from all households; WRA from 9 households selected randomly from the PSC households; SAC from 6 of the 9 WRA households; men from 4 of the 6 SAC households. Based on estimates of the numbers of each demographic group per household, the likely sample size within each demographic group was estimated to be approximately 1459 PSC, 750 WRA, 762 SAC, and 252 men. These sample sizes were estimated to provide adequate statistical power to detect changes in a range of nutritional indicators for PSC from previous MNS, including vitamin A deficiency, anaemia, zinc and iron deficiency, and stunting, based on 90% household and individual participation^13,29,30^. In each of the target population groups, individuals deemed ineligible to participate in the MNS included those^1^: chronically ill^2^; outside the target age groups^3^; diagnosed with chronic disease or long term illness requiring treatment^4^; with acute or chronic infection which may alter serum zinc status^5^; on regular prescribed medicine^6^; using oral contraceptive pills or hormone replacement therapy^7^; regularly using dietary supplements including iodine, zinc or selenium supplements^8^; children with a physical disability that would affect either height or weight measurement. These exclusion criteria were based on the requirements of the MNS and not for the purposes of studying plasma Se status. Survey participants identified as having severe anaemia (hemoglobin <7 g dL^−1^), malaria (based on positive results from a rapid diagnostic kit), moderate or severe acute malnutrition (using age-appropriate mid- upper arm circumference (MUAC) cutoffs), haematuria (based on urine dipstick in all groups except WRA), or severe illness as identified by a team nurse, were referred to a local hospital for evaluation.

### Data and sample collection

The MDHS and MNS teams visited clusters sequentially; the MDHS collected data electronically using tablet computers; the MNS used a paper questionnaire. The tablets were programmed so that MDHS teams were notified of eligible households and demographic groups for the MNS. For each eligible household, an MDHS team placed a household label with a unique barcode on the questionnaire and entered the barcode number into the tablet. This barcode number enabled data collected independently by the MDHS and the MNS teams to be linked. Once MDHS data collection was completed within a cluster, the questionnaire booklets were given to the MNS team. The MNS team would typically proceed to a cluster within 2–3 days of the MDHS data collection.

The MNS team visited each household that had indicated to the MDHS team that they were willing to participate in the MNS. The head of each MNS household was asked for written consent so potential participants from that household could be issued with uniquely barcoded bracelets, linked to the questionnaire booklet, and accompanied by MNS staff to the field laboratory. Individual interviews and specimen collection was then conducted, subject to further individual consent from the individual or parent/guardian^13,29,30^. If eligible households, or individuals within households, were unavailable, up to two follow-up visits were made to each household; households were not replaced.

Whole blood venous samples (~5 mL) were collected into trace-element free purple top vacutainers through venipuncture, using a butterfly needle, by experienced phlebotomists. All individuals (PSC, SAC, WRA and men) were seated prior to blood collection, skin was wiped with 70% alcohol at the area of antecubital vein, and the arm was restricted with a tourniquet for maximum of 1 minute. During blood collection, the tourniquet was loosened before removing the needle from the arm. Once blood was collected, the vacutainer was inverted 10 times to allow blood to mix with the EDTA in the tube to prevent clotting. Blood samples in vacutainers were centrifuged at 3,500 rpm for 10 minutes in mobile centrifuges, and plasma was aliquoted into sterile labeled cryovials; ~3 mL was dispensed into transparent, flat-based 8 mL polypropylene tubes (57 × 16.5 mm; Sarstedt, Nümbrecht, Germany), capped using transparent high-density polyethylene, and racked into groups of 50. The samples were stored in portable freezers at −20 °C, transferred to District-level hospital storage, and then to the central laboratory at Community Health Sciences Unit (CHSU), Lilongwe, for storage in boxes of 100. The plasma samples were transferred on dry ice for Se analyses, to the University of Nottingham, United Kingdom.

Strict quality control measures were followed during blood collection and transportation using well-trained nurses/clinicians and laboratory technicians under the supervision of the Centers for Disease Control & Disease Prevention (CDC) technical team. At every stage of sample collection, laboratory technicians ensured that all containers for the collection and transportation of blood samples were clean and free from any contaminants and interfering substances. The health professionals involved in blood collection followed strict sterile procedures and medical ethics. Throughout the sample collection, transportation, analysis, and disposal, all blood samples were treated as potentially infectious for HIV and other infectious agents, and disposable polyethylene powder free gloves were used for blood handling.

### Analysis of blood plasma for Se

The concentrations of Se in plasma samples were measured using inductively coupled plasma-mass spectrometry (ICP-MS; Thermo Fisher Scientific iCAPQ, Thermo Fisher Scientific, Bremen, Germany). Samples were introduced from an autosampler incorporating an ASXpress™ rapid uptake module (Cetac ASX-520, Teledyne Technologies Inc., Omaha, NE, USA) through a perfluoroalkoxy (PFA) Microflow PFA-ST nebuliser (Thermo Fisher Scientific, Bremen, Germany). External Se calibration standards at 0, 5, and 10 ng mL^−1^ (SCP Science ‘PlasmaCal’; certified concentration 1003 ± 6 µg mL^−1^) were prepared in 0.5% HNO_3_ (trace analysis grade (TAG), Fisher Scientific UK Ltd, Loughborough, UK). Samples were diluted 1 in 31 in 0.5% HNO_3_ (TAG). The ICP-MS was operated in ‘collision-reaction cell mode’, with kinetic energy discrimination, using H_2_ as the cell gas to maximise the sensitivity for Se determination. Correction for drift was undertaken using three internal standards (^72^Ge, ^103^Rh, ^185^Re in 0.5% HNO_3_ and 4% methanol) introduced to the sample flow via a Y-piece; methanol enhances Se ionisation in the plasma. The limit of detection (LOD) for Se was 0.0011 ng mL^−1^, representing 3× the standard deviation (SD) of 10 operational blanks. Accuracy was verified by the use of appropriate reference materials (Seronorm-1 and Seronorm-2; Nycomed Pharma AS, Billingstad, Norway). Mean recoveries were 96.5% (SD = 2.3%) and 96.9% (SD = 2.9%) for Seronorm-1 (Lot 1309438; accredited value 86.5 ng/mL) and Seronorm-2 (Lot 1309416; accredited value 137 ng mL^−1^), respectively (n = 47). All plasma samples with Se concentrations >200 ng mL^−1^ (n = 27) were re-run for confirmation; the mean difference was 3%, excluding one outlier. Samples were also analysed for gadolinium, used in MRI scanning, to confirm that the doubly charged species (^156^Gd^++^; m/z = 78), presented a negligible interference to the analyte ^78^Se.

### Data analyses

In this paper, we report the analysis of plasma Se concentrations for PSC, SAC, WRA, and men. We focused our spatial analysis on WRA because thresholds linked to optimal activities of selenoproteins glutathione peroxidase 3 (GPx3) and iodothyronine deiodinase (IDI), respectively are only applicable to adults^9^, and due to the greater numbers of WRA in the sample. Summary statistics for WRA plasma Se concentration data are shown in Supplementary Information. Given the marked skewness of these data; these were transformed to natural logarithms, and the summary statistics on this scale are also shown in Supplementary Information. These log_*e*_-transformed data were used for the remaining analysis.

As described above, the sampling units are individuals from within households selected at random within clusters. This sampling structure was accounted for in the analysis with a linear mixed model (LMM) in which the only fixed effect was a constant mean. The variation in plasma Se concentration between individuals within households (which includes analytical error) was treated as a random effect of mean zero with homogeneous variance over all households. The between-household within-cluster variation was also treated as an independent random effect of mean zero and with homogeneous variance over all clusters. The between-cluster variation was modelled as a spatially correlated second-order Gaussian random variable with a Matérn correlation function^31^. Exploratory analysis of the mean values for the clusters suggested that the assumption of stationary variation about a fixed mean was reasonable, with no pronounced trends. It also suggested that it was reasonable to treat the spatial dependence of the cluster means as isotropic (i.e. dependent only on the distance between any two clusters and not on the direction of the vector that joins them). The variance components, and the parameters of the Matérn correlation function were estimated by residual maximum likelihood (REML). Following the recommendation of Diggle and Ribeiro^32^, the smoothness parameter of the Matérn correlation was estimated by the profile likelihood.

The between-individuals (within-households) variance (log variable) was 0.028 and the between-household (within cluster) variance was 0.018. The between-cluster variance was much larger (0.15). The smoothness parameter estimated by the maximum profile likelihood (equivalent to the familiar exponential correlation function) was 0.5 and the spatial parameter was 39.4 km which means that the variation between cluster means shows spatial dependence up to a distance of about 120 km. To validate this model, each observation was predicted from the rest by cross-validation. The assumption of normally distributed cross-validation errors was plausible (Supplementary Information), and the standardised squared prediction error of the cross-validation predictions had a median value of 0.33 which falls within the 95% confidence interval for this statistic under prediction with a valid covariance model and normal errors^33^.

To map plasma Se concentrations for WRA, ordinary kriging estimates of individual concentrations were computed at nodes of a 500 × 500 m square grid. At each node, the probability was computed that the true value of this variable took values below thresholds for optimal plasma Se <84.9 and <64.8 ng mL^−1^, the lower thresholds linked to optimal activities of the selenoproteins GPx3 and IDI, respectively^9^. This probability was obtained from the prediction distribution of log-transformed plasma Se concentration at each location on the grid. This is a conditional distribution, dependent on the fitted model and the neighbouring observations. The ordinary kriging (OK) prediction at each grid node was treated as the mean value of the prediction distribution and the OK variance as the prediction variance. The OK predictions of plasma Se concentration were back-transformed from the log scale by exponentiation which gives a median-unbiased central value of the prediction distribution on the original scale^34^. The probabilities of falling below the thresholds were mapped using interval values based on a verbal scale for communicating uncertain information, supplemented with numerical values in the legend, following Lark *et al*.^35,36^.

## Supplementary information

Supplementary Information

## Data Availability

The datasets generated during the current study are available from the corresponding author on reasonable request.
