# Supplementary material for: The risk of selenium deficiency in Malawi is large and varies over multiple spatial scales
Source: Sci Rep. 2019 Apr 25;9:6566. doi: 10.1038/s41598-019-43013-z (PMC6484074; doi:10.1038/s41598-019-43013-z)
Supplement: Supplementary file 1 — Supplementary Information [file 41598_2019_43013_MOESM1_ESM.docx]

Supplementary Information

The risk of selenium deficiency in Malawi is large and varies over multiple spatial scales

Felix P. Phiri, E. Louise Ander, Elizabeth H. Bailey, Benson Chilima, Allan D.C. Chilimba, Jellita Gondwe, Edward J.M. Joy, Alexander A. Kalimbira, Diriba B. Kumssa, R. Murray Lark, John C. Phuka, Andrew Salter, Parminder S. Suchdev, Michael J. Watts, Scott D. Young, Martin R. Broadley

Corresponding authors:

Felix Phiri and Martin Broadley

Email: felixphiri8@gmail.com and martin.broadley@nottingham.ac.uk

This PDF file includes:

Figs. S1 to S2

Table S1

Fig. S1. Frequency distributions of Se plasma concentration among women of reproductive age (WRA) on the (a) original, and (b) natural logarithmic scales.

Fig. S2. Cross-validation errors based on predictions of each observation from the rest (1).

Table S1. Summary statistics for plasma Se concentration among women of reproductive age (WRA) on the original and logarithmic scales.

| Plasma Se concentration | Mean | Standard Deviation | Median | Min | Max | Skewness |
| --- | --- | --- | --- | --- | --- | --- |
| ng mL^-1^ | 83.72 | 38.38 | 78.36 | 11.05 | 374.44 | 2.62 |
| log ng mL^-1^ | 4.34 | 0.40 | 4.36 | 2.40 | 5.93 | –0.02 |

References

1. Lark RM (2000) A comparison of some robust estimators of the variogram for use in soil survey. *Eur J Soil Sci* 51: 137–157.
